# Mapping the Autistic Advantage from the Accounts of Adults Diagnosed with Autism: A Qualitative Study

**DOI:** 10.1089/aut.2018.0035

**Published:** 2019-04-13

**Authors:** Ginny Russell, Steven K. Kapp, Daisy Elliott, Chris Elphick, Ruth Gwernan-Jones, Christabel Owens

**Affiliations:** ^1^ College of Medicine and Health, University of Exeter Medical School, University of Exeter, Exeter, United Kingdom.; ^2^ College of Sociology, Philosophy and Anthropology, Social Science and International Studies, University of Exeter, Exeter, United Kingdom.

**Keywords:** autism, autistic intelligence, thematic analysis, strengths and weaknesses, neurodiversity

## Abstract

***Background:*** Autism has been associated with specific cognitive strengths. Strengths and weaknesses have traditionally been conceptualized as dichotomous.

***Methods:*** We conducted 28 semi-structured interviews with autistic adults. Maximum variation sampling was used to ensure diversity in relation to support needs. We asked which personal traits adults attributed to their autism, and how these have helped in the workplace, in relationships, and beyond. Data were collected in two stages. Responses were analyzed using content and thematic techniques.

***Results:*** The ability to hyperfocus, attention to detail, good memory, and creativity were the most frequently described traits. Participants also described specific qualities relating to social interaction, such as honesty, loyalty, and empathy for animals or for other autistic people. In thematic analysis we found that traits associated with autism could be experienced *either* as advantageous or disadvantageous dependent on moderating influences. Moderating influences included the social context in which behaviors occurred, the ability to control behaviors, and the extent to which traits were expressed.

***Conclusions:*** Separating autistic strengths from weaknesses may be a false dichotomy if traits cannot be isolated as separate constructs of strengths or deficits. If attempts to isolate problematic traits from advantageous traits are ill conceived, there may be implications for interventions that have reduction in autistic traits as a primary outcome measure.

**Lay Summary:**

**Why was this study done?**

The study was done to find out what autistic adults could tell us about their own abilities. They told us about their abilities and how these abilities had helped them in their everyday lives: at work, in their relationships with other people, and at home.

**What was the purpose of this study?**

To tell a story about what aspects of their autism adults thought were of benefit, when going about their daily lives.

**What did the researchers do?**

The researchers interviewed 24 adults who had an autism diagnosis. Some lived in residential care and others lived alone in rented apartments. Some people were interviewed twice. Most people said they enjoyed the experience of being interviewed.

Once the interviews were done, they were typed up and the researchers tried to figure out what were the common themes over all the stories they had heard. They thought about the themes, then did some more interviews with autistic adults to check they were on the right lines. After discussing them, they wrote the story.

**What were the results of the study?**

Hyper focus, attention to detail, and the ability to remember were the abilities that autistic people said benefitted them most often. But autistic adults who were interviewed said although their autistic traits were sometimes helpful, at other times they hindered their progress. So the same trait might be useful in some circumstances and unhelpful in other situations. For example, hypersensitivity led one person to enjoy nature, but was difficult to cope with in crowded streets. The study highlights this interchangeability.

**What do these findings add to what was already known?**

Before, autistic people were known to have both strengths and challenges, but studies tended to separate autistic strengths and weaknesses as different things. We theorize that some traits are expressed as behaviors that may serve to improve or hinder autistic people's progress, but this depends on their situation (context).

**What are potential weaknesses in the study?**

Because the researchers used interviews, they did not include any nonverbal autistic people in the study.

**How will these findings help autistic adults now or in the future?**

It could be useful to think about autism in a way that does not focus on deficits and this study will help us to do that. Plus, if an autistic trait can give people an advantage or a disadvantage, interventions aimed at reducing autistic behaviors might risk dampening advantageous traits as they seek to help with difficulties. That means, autistic adults and children might lose useful abilities when and if they are treated for traits that can also be problematic. The researchers hope their study will lead to more discussion about these types of ideas.

## Introduction

Activists and scholars have called for autism research to focus on skills (as opposed to deficits).^1,2^ Indeed, most medical literature is focused on autistic impairments, but this may harm autistic individuals' identity construction and well-being.^3^

Autistic adults have created an alternative narrative that focuses on autism as an integrated identity that brings valuable traits as well as challenges.^4–7^ The neurodiversity movement positions acceptance and legal protection for neurological differences (e.g., autism) alongside those for differences in race, religion, and sexuality,^8^ a “difference not deficit” perspective that acknowledges weaknesses and support needs.^9^ However, others involved in building counter-narratives to medical deficit-based descriptions of autism tend to dichotomize strengths and weaknesses as separate entities, describing autistic people as having deficits in some areas and strengths in others.^7^

The scientific literature also largely discusses strengths and deficits of autism as distinct. In 2009, a conference article by autistic researcher Dawson reviewed 71 studies listing 52 reports of autistic cognitive strengths,^10^ although 29 reported or interpreted at least one finding as a deficit.^10,11^ Most of the studies reviewed ulitized cognitive tests, like the Embedded Figures Test^12^ and the Block Design nonverbal IQ subtest.^13^ Tests have found strengths in perceptual abilities such as pattern recognition,^14^ language, and social information processing for autistic individuals,^10^ yet these tests largely examine cognition in artificial settings that lack ecological validity. This is problematic when unpacking how advantageous traits impact autistic adults' everyday lives.

Older studies dismissed abilities in autistic individuals as “islets of ability.”^12^ More recently, findings have given rise to the notion of “autistic intelligence,” described as a qualitatively different form of intelligence that relies less on verbal comprehension than standard IQ tests.^15^ Yet strengths-based measurements are prey to similar critiques as IQ tests, that is, they are designed and implemented by a normative framing of what constitutes strengths.

Scientific literature has influenced professional and lay accounts of autism as involving isolated strengths. The idea of separate cognitive strengths that autism confers has also been taken up in popular science writing, for example, the *New Scientist* described “the autistic advantage.”^16^ Professionals have produced accounts that describe *only* the positive features of autism. For example, a recent infographic aimed at educators encapsulates positive aspects of autism, including attention to detail, focus, and retention of facts.^17^

Evidence of autistic strengths has also come from narratives by autistic people. Autobiographical material from autistic authors analyzed together with interview data showed that the majority of authors reported exceptional abilities (in maths, music, or art).^18^ Analyses of autobiographies and online accounts of autistic adults report similar results.^19–21^ Such activities, show these autistic adults are able to communicate in sophisticated ways on their own terms, so may inherently omit the perspective of more impaired individuals. Across the spectrum, autism is notoriously heterogeneous in presentation: people with a diagnosis range from those with severe intellectual disability who require full-time care, to postgraduate students and professionals who live independently, or from verbally fluent individuals with idiosyncratic language to nonverbal individuals.^22^ Including perspectives from across the whole spectrum remains an ongoing research challenge.^23^

Anecdotal accounts from autistic adults outside of the academic literature suggest that many have attained rewarding work, in part, because of their enhanced perception or perseverative abilities.^24–26^ These reports hint at a positive impact of atypical processing, perception, and cognitive abilities on daily life.

Advocates and disability study scholars have offered a more political critique of the concept of autistic advantage. Powerful arguments have been made about how the idea of “advantageous” or “disadvantageous” exists only in relation to the values of neoliberal society.^27^ Self-advocates have made strongly inclusive statements about what does and does not get considered valid communication and intelligence.^28^

If aspects of autism can be beneficial, advantages may be lost if interventions seek to minimize autistic traits in a blanket way. Thus the topic of strengths is wrapped up with the topic of treatment, in that the autism community has expressed concerns that treating “autism” as a generic target may lead to the elimination of strengths that the condition may bring.^29^ Researchers have shared the concern that a cure for autism might dampen useful abilities,^30,31^ offering models,^32,33^ and sharing research^34^ that intertwine strengths and disability. One case study^35^ described therapy that led a 6-year-old autistic girl to develop a range of functional behaviors but to lose her exceptional drawing skills. In another example, a group who no longer met criteria for an autism spectrum diagnosis after intensive treatment showed typical levels of auditory discrimination, whereas peers who continued to meet criteria demonstrated enhanced pitch discrimination (a strength associated with autism).^36^

We examined first-person accounts, which allowed us to gather evidence on which abilities autistic adults themselves report as advantageous, to provide a holistic picture of how their abilities have aided them in everyday life.

## Methods

We took a critical realist stance^37^ that our participants' accounts were filtered through their own interpretations, and we made meaning of these through our core concepts. We derived these from the thematic analysis approach of Braun and Clarke,^38^ who write, “We do not subscribe to a naïve realist view of qualitative research where the researcher can simply ‘give voice’” (p.7). Our aim was to examine to what extent (if at all), and in what ways, autistic adults described their autism as advantageous. Our team included one autistic researcher, two methodological experts, and two others who described themselves as non-neurotypical. Our combined expertise lies in psychology, anthropology, and sociology.

### Sample

The sample consisted of 24 adults who had received a clinical diagnosis of an autism spectrum disorder. Participants had to have a clinical autism diagnosis on their medical records to attend the centers, groups, and homes through which we recruited. We used a maximum variation sampling approach to elicit a full range of experience. The Diagnostic and Statistical Manual of Mental Disorders (DSM-5) diagnostic criteria specify autism severity criteria.^39^ The planned sample had three levels organized around levels of support received:

More than 25% of sample receiving low-level support (living independently).
More than 25% receiving mid-level support (having a live-in carer).
More than 25% receiving high-level support (living in full-time residential care).

The small subsample sizes neither allow for comparative analysis between the groups nor were theoretically justified. The reason we used housing status as a proxy for support was pragmatic. The housing setting, combined with participants' support around their daily living, provided a rounded measure of autonomy and was easy to gauge because the setting was the recruitment route.

### Recruitment and ethics

We recruited in South West England through residential homes specializing in housing autistic adults and through two National Autistic Society (NAS) skills-training centers. The NAS granted access to the centers and to some of the homes they run; a local support service granted access to another residential home. We met with liaison staff members in each site, who knew the service users well, and we invited participants to be interviewed only after careful consultation with the staff in each setting. In addition, we recruited participants through U.K.-based autism groups, with the project website putting out a call for adults with a diagnosis of autism.

The Exeter University ethics committee for Social Sciences granted ethical approval. Before interviews, we described the study aims, the voluntary nature of participation, and how data would be used to each participant. We provided participants with written information sheets, which we read aloud, detailing the above, the aims of the study, and our contact details. Participants who wished to be interviewed signed written consent forms. Participants who did not have mental capacity to give informed consent were not included. We judged this in consultation with carers.

### Data collection

We collected data in individual face-to-face semistructured interviews, giving the option of instant messaging or email interviews for three participants who were geographically distant. Three researchers (C.E., D.E., and G.R.) conducted all interviews. Face-to-face interviews, carried out as part of a wider study on autistic adults' experiences, always took place in a dedicated quiet room, with an option to have a carer or service provider present. The topic guide (Appendix 1) was a loose guide for interviewers, enabling them to steer conversation around questions of interest; they did not repeat the guide verbatim. Instead, interviewers were reactive to the nuances of each participants' abilities and situation.

We collected and analyzed data in two stages. At the first stage, we interviewed 13 participants and transcribed data. After this, preliminary analysis identified candidate themes. We then conducted a second sweep of data collection, 7 months after the first collection period, following recommendations to carry out at least two data sweeps, with a time gap between sweeps.^38^ Stage 2 interviews asked more targeted questions about emerging themes. For example, at Stage 1, participants talked about advantages but also spontaneously about challenges. This was followed up in Stage 2. Four Stage 1 participants were interviewed again in Stage 2 at their own request. The researchers aimed to emulate the researcher–participant relationship of “good guests” described by Abbott.^40^ Interviewers emphasized the value of contributions being made, as well as being alert to changes in the conversational tone for signs of reticence, disinterest, or misunderstanding.

### Content analysis

The transcribed text from interviews was first imported into NVivo software to support coding and data management. Two researchers (G.R. and C.E.) read through the interviews to code traits that participants described as advantageous. We conducted a summative content analysis that involved counting and comparisons of keywords in content, and the underlying context.^41^ We counted references to the traits and abilities participants attributed to their autism and experienced as advantageous. All words used to conceptualize such traits were extracted. Instances were nested in the wider text to check the context of usage. A word cloud was then created where the size of the word was proportional to the frequency of usage in the data.

### Thematic analysis

Two researchers (G.R. and C.E.) familiarized themselves with the data from five interviews and developed a consensus coding frame. We generated a combination of theory-driven (deductive) and data-driven (inductive) codes. Codes were then sorted to identify potential patterns or candidate themes. Candidate themes were reviewed again after Stage 2 to check whether there were enough data to support them, and that they were a coherent reflection of the whole data set. A thematic map was constructed by moving themes around a board and identifying the relationships between them. Once a satisfactory account of the data was agreed, the analytic narrative was written. A great deal of unprompted talk took place during the interviews, and our analysis reflects this.

## Results

We conducted 28 interviews and had 24 participants. Table 1 gives details of their autism diagnoses and other demographic information. They were fairly evenly divided between support categories. Fifteen participants were unemployed, six were employed, and three were students. Six had no educational qualifications, 11 left school with some qualifications but did not progress with their education, and 7 had degrees and/or some form of higher education. Six participants chose to have a carer or parent present during interviews. Participants reported a variety of co-occurring conditions, including obsessive compulsive disorder and dyspraxia. The mean duration of interviews was 59 minutes.

**Table 1.** Participant Demographic Information

	*Participant age*	*Gender*	*Autism diagnosis*	*When received diagnosis of autism (age, if known by participant)*	*Support received*	*Interviewed twice?*
P1	28	Male	Autism	Child (8)	Low	
P2	65	Male	Asperger's	Adult (48)	Mid	
P3	33	Male	Asperger's	Child (11)	Low	Yes
P4	27	Male	Autism	Child	High	
P5	27	Male	Autism	Child (13)	Mid	
P6	28	Male	Asperger's	Child	Mid	
P7	55	Male	Autism	Adult (44)	Low	
P8	49	Male	Autism	Adult (28)	High	Yes
P9	26	Female	Asperger's	Adult (24)	Mid	
P10	30	Female	Asperger's	Child (11)	Low	
P11	56	Female	Asperger's	Adult (43)	Low	Yes
P12	23	Male	Asperger's	Child (15)	Low	
P13	41	Male	Autism	Adult (21)	High	
P14	56	Male	Autism	Child (5)	High	
P15	45	Female	Autism	Adult (30)	Mid	
P16	21	Female	Autism	Child (11)	Mid	
P17	48	Female	Autism	Adult (44)	Low	
P18	38	Male	Autism	Adult (31)	Mid	
P19	32	Male	Asperger's	Adult (30)	Mid	
P20	37	Female	Asperger's	Adult (34)	Mid	Yes
P21	48	Male	Autism	Adult (33)	High	
P22	36	Male	Asperger's	Child (17)	High	
P23	21	Male	Autism	Child (4)	High	
P24	54	Male	Asperger's	Adult (48)	Low	

### Content

**FIG. 1.**
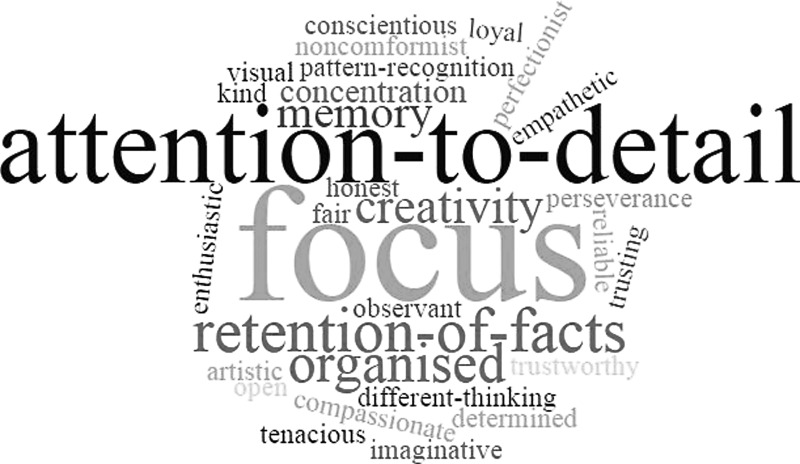
Word cloud showing advantageous traits as described by participants.

All but one participant was able to describe their own traits and how these had benefitted them, and the majority of participants could and did attribute these to autism. Traits included perceptual differences, memory, focus, and attention to detail, logic, and vivid imagination. Frequencies of word occurrence were represented in a word cloud (Fig. 1). Not all participants reported all of the traits illustrated in Figure 1. Some participants actively denied having a trait that others described themselves as having (e.g., “my memory really isn't that good”; “I regret showing reduced empathy”).

### Themes

Three themes were identified in participants' accounts: (1) experience of difference, (2) false dichotomies, and (3) moderating influences.

#### Theme 1: Experience of difference

This theme encompassed how participants conceptualized the causes of their difference from “neurotypical” (NT) individuals. Some participants recounted having autistic differences in perception, or “wiring,” and others felt although they differed from NT people, this was not attributable to autism. A few interpreted their experiences in terms of autism theories they knew about, for example, “theory of mind was the problem” (P10) or “due to my weak central coherence” (P2). Overall, most participants talked about autism as a set of qualities they possessed rather than an illness they had.

Many participants described their differences as stemming from a difference in the way their mental abilities were configured or how they processed or perceived the world. Examples included graphic descriptions of exceptional memory as a “small tape recorder in my head” (P4), “I remember conversations word-for-word” (P10), and “I'm replaying it in my mind and I'm back there” (P16). Hyperperception of color and shape was described, which was linked to attention to detail or pattern recognition:

If you watch a film the colour goes green, red, light blue, yellow … The first thing your eye will be drawn to is the colour and it just continues, it just walks you through the film and continues …It's beautiful. (P24)

Such perceptual and processing differences were implied as causes of cognitive or behavioral traits associated with autism.

Data gave the impression that some autistic people can do things other people cannot, and that they cannot do things other people can. Traits that participants described (Fig. 1) fell into two broad categories, namely cognitive/perceptual abilities and social skills.

In the cognitive/perceptual abilities category, ability to focus was mentioned by almost all participants, variously described as “like zooming in” (P20), “unwavering focus” (P1), “hyperfocus” (P11), and “focusing on one thing” (P20). “Focusing on it and focusing and focusing” (P9).

When considering advantages of the participants who were employed or at university, many described their ability to “concentrate” or “hyperfocus” on one task to the exclusion of all else as a huge benefit. This skill was described as tenacity or perseverance but also meant the exclusion of attention from other people's priorities, or interests that might be considered more typical:

Perseverance—not giving up. Like I'd much rather have worked on my maths homework than go for a party. It was my 18th party … and my mum organised the party. And it was maths homework night, a Thursday night, and I had to hand it in the next day. So I stayed at school as long as I could ‘til the school closed at 6 o'clock that night so I could get my maths homework done. It was more important than having a party. (P11)

A young man who was studying for a postgraduate degree described how such unwavering focus was beneficial to him in academic life:

I can … sit down and during those timetabled hours [and] work with little movement away from the task … I believe the unwavering focus to a subject has aided me academically, my different approach to thinking has also aided me in this way and of course I wouldn't have my job without it and the comfortable life. (P1)

Participants were often able to describe how attention to detail had aided them at work:

Because with attention to detail, obviously outside of the house, in work, I've managed to sort of win customer service awards. And without my attention … you know, without having the attention-to-detail ability I wouldn't have been able to obviously spot the things that obviously other people wouldn't. (P5)

This participant's supermarket job had involved shelf stacking and getting items exactly “in the shape” (P5). He had won a prestigious customer service award and commented on his own abilities, “I am able to notice the colours and everything on the shelf” (P5).

One striking difference within individuals' accounts was the sense of flow in conversation when on their own topic of interest, in marked contrast to frequent stumbling and incoherent speech facing reflexive questions.

Several participants described strengths in their social skills, describing themselves as compassionate and empathetic toward animals or “for others on the spectrum” (P24). One participant commented, “I can pick up traits and know why they're happening potentially, and people can have a bit more trust in me for knowing that's the case” (P12). Others mentioned how autism meant they “forget social norms completely” (P9) and experienced “not needing to conform” (P10).

#### Theme 2: False dichotomies

Participants gave accounts of traits as advantageous and simultaneously disadvantageous in the workplace, in relationships, and at home. There was no boundary between a strength and a weakness. There were many instances of a trait that was described as advantageous simultaneously causing problems with physical or mental health, including loss of sleep, and difficulties for other people. Attention to detail, for example, could have a negative outcome, especially if associated with switching tasks. One participant who worked as a gardener talked about her attention to detail when weeding, allowing her to complete tasks to a high standard, but that her perfectionism could be problematic with time constraints. One participant described how such traits could be “stressful to work with” (P24).

Honesty, reliability (when anxiety did not intrude), integrity, and a hatred of lies were all attributed to autism: an “extreme sense of justice” (P10) as one participant put it. One described “two sides” to this: being honest with people, and open, therefore people like you, but metaphorically “dropp[ing] a brick when refusing to express an appropriate white lie” (P2).

Our topic guide asked participants which of their abilities they attributed to autism. Several participants had difficulty in separating what was attributable to “autism” from what was attributable to “themselves,” and in imagining what life would be like without autism. One participant was “confused about what is autism” (P23). The participants who made the point explicitly that there is no “autism—self” opposition thus saw their abilities and skills more holistically as generalized personality traits, which included autistic traits:

Really though I don't attribute anything about me to being autistic, it's just me. I can't answer the question properly because I am me, including being autistic. (P15)

Our analysis showed that some participants had difficulty parceling off aspects of their behavior as “autism,” again, the self/autism division seemed a false dichotomy.

#### Theme 3: Moderating influences

Factors that might determine whether a participant experienced a trait as advantageous or disadvantageous were classified as moderating influences. Categories were the moderating influences of social context, controllability and extent, and perspective.

##### Social context

Coding highlighted different social and environmental contexts in which a trait might play out as either a benefit or a hindrance. One participant referred to the pluses and minuses of hypersensitivity. She described a “being able to experience things more intensely, such as art or nature, even though sensory sensitivities can be awful at times” (P15). Social situations that prompted such comments included a crowded street, a busy restaurant, and a highly interactive workplace. Another participant talked about how a lack of empathy could be useful in the army, and described how sticking to routines had been a positive attribute in prison because routines were mandatory.

##### Controllability and extent

Problems or benefits were moderated by the extent to which individuals felt they were “overdoing it.” Some described a feeling of being “compelled” (P9) to complete a task in one sitting, or feeling obliged to “go all the way down the rabbit hole” (P1), resulting in mental health issues, such as anxiety about not completing tasks, and physical health issues, including lack of sleep:

The hardest thing to me [is] until it's done, it consumes my life. When I was younger this was an issue especially with work—I'd sometimes move into the library for 72 hours to write an assignment. (P1)

Social skills attributed to autism, such as openness, were also experienced as beneficial, but became a problem when overexpressed or when taken too far: “I am too open with things” (P11).

Three participants had co-occurring diagnoses of obsessive compulsive disorder and described how problems arose when special interests on which they were able to focus became “obsessions” and started to control them: “I don't actually want it to happen” (P21). One participant had suffered a chronic wrist injury from overplaying the guitar, and described feeling “compelled” (P9) to learn to play. However, there was talk of a real benefit, including experiencing “joy” (P3, P9, P12) and “flow” (P11, P18, P24), when participants felt they were in control.

##### Perspective

Finally, some participants pointed out that advantages or disadvantages were in the eye of the beholder:

Why is obsession bad and the ability to focus on something that you like [good] … Why was Sir Isaac Newton bad when he was so obsessed about that apple falling from that tree? (P3)

The participant who worked at a supermarket stacking shelves, and described how preoccupation with the objects that needed sorting was advantageous in his work, although could be considered a “preoccupation with unusual objects” as mentioned in DSM-5.^39^

We found that what a medical practitioner might consider impairment could sometimes act to an autistic person's advantage. One participant spoke about how “some of your weaknesses are our strengths” and illustrated the point with his experience as a teaching assistant, using lunchtimes to focus on “sorting out stuff” for the children when others were “just gossiping” (P11). What other teachers framed as a deficit (“you might say I'm not friendly/sociable” [P11]), he recast as an advantage.

## Discussion

The skills most frequently described tallied with those reported in previous accounts:^16,17^ hyperfocus, attention to detail, good memory, and creativity were associated with autism bringing context-dependent advantages. Traits such as empathy for others and/or for animals and creativity have been evidenced elsewhere in autistic individuals.^42–44^ Not all participants reported all the traits listed, and there was considerable heterogeneity in abilities. Almost all could describe some advantageous “autistic” traits, suggesting beneficial characteristics may not be isolated to a small section of the population of people on the autism spectrum.

**FIG. 2.**
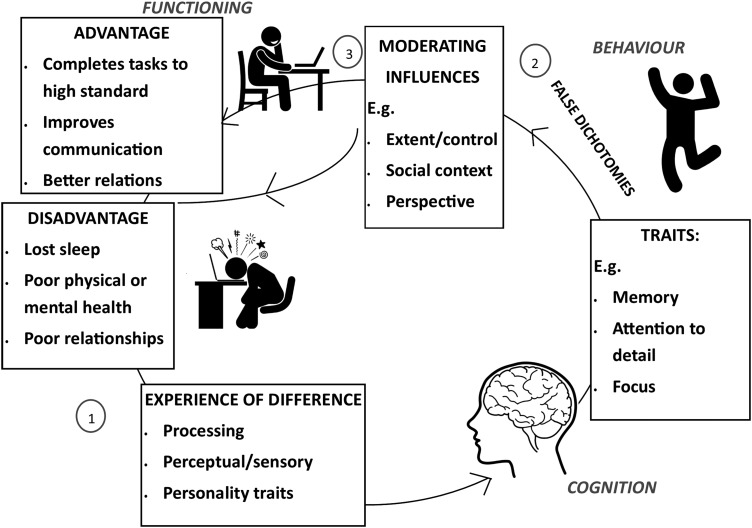
A thematic map.

Participants often described advantages that were the flip side of their autistic “impairment.” Our analytic map (Fig. 2) suggests that the traits participants reported could act either as advantageous or disadvantageous, dependent on the context, including circumstance, perspective, and the extent to which they were under an individual's control. So, for example, focus might result from the same characteristic that underpins not being able to switch from topic to topic defined as a problematic symptom in DSM-5: “difficulties with transitions”^41^ (p.50). This recasting of traits pathologized by diagnostic criteria as advantageous in some circumstances dovetails with emerging literature on how aspects of neurodevelopmental conditions like autism can function to a person's adavntage.^42–44^

If isolating problematic traits from advantageous traits is misguided, interventions that have reduction in symptoms of autism (i.e., behavioral traits) as their primary outcome measure, for example, the “optimal outcome,”^45^ must take account, in case interventions unintentionally dampen advantageous traits. Some have argued that medical research should target underlying neurodevelopmental mechanisms that produce distressing symptoms.^46,47^ Our data indicate it may be difficult to eradicate troubling challenges attributable to “autism” without at the same time losing valuable aspects.

Figure 2 illustrates how accommodation strategies are promising avenues to engender advantages. Our finding that social context moderates advantages, for example, suggests that autistic people can make significant contributions to society and flourish in the right social environment. It also suggests that when traits are overexpressed or out of control, they become disadvantageous, so inhibition and self-control strategies could be helpful.

The concept of the “autistic advantage” might be usefully applied in fostering a positive self-identity.^48^ According to many, identifying autism can have various positive effects, in addition to gaining access to services, through reducing self-criticism and fostering an identity.^3,49^ That is true if autism really is considered as a positive self-identity. Clinicians may “inadvertently promote negative stereotypes, diminish patients' self-worth, and portray them as broken individuals or burdens to others” (p.505).^50^ Ascribing a medical diagnosis of “disorder” inherently dichotomizes who is sick or unhealthy and who is well.^51^ Reporting accounts of traits that can be beneficial may help to foster a more rounded vocabulary in autism discourse for clinicians, autistic individuals, and their families.

Illness narratives have often been described as “gift” or “tragedy” narratives.^52^ Autism is viewed by society, by medics, and by many parents as a problem (a tragedy narrative). Participants negotiated the gulf between *autism-as-gift* and *autism-as-tragedy* through their life stories showing how traits were both helpful and a hindrance. The experience of knowing you have autism, according to conventional wisdom, means there is something wrong with you. However, our participants experienced autism as integral to themselves, for better or for worse. A way to square the resulting dissonance was for participants to allow that autistic traits could cause them problems, but could confer benefits too. In this way, autistic participants negotiated their understandings of self without dichotomizing their biographies into either “gift” or “tragedy” narratives.

Narratives about autism clearly vary culturally, and between individuals and settings.^53^ Bringing advantageous aspects of autism to the fore could be helpful to construct (as well as report on) a more nuanced narrative, destigmatizing autism. Were a more rounded model of neurodevelopmental difference to be incorporated into medical texts, would this negate the need for diagnosis and treatment? The problem with medical diagnosis is it necessarily identifies autism as a condition needing remediation—which can be inherently stigmatizing. But diagnosis is also needed both for access to services and as a rallying point around which activists can mobilize.^54^

The findings make us wary of describing autistic advantages as fixed traits, rather their expression (and development) is context dependent. Individuals who do not think they live up to or are not perceived as living up to a fixed reconceptualization of strengths might feel devalued. Furthermore, several participants struggled to attribute traits to their “autism,” perhaps because they do not experience autism as a separate entity—unlike most researchers' descriptions. Autism may be a group of multidimensional traits experienced as a holistic aspect of selfhood. We recognized that the dichotomies we set up in our questions between autism and self, and strengths and weaknesses, were largely false.

Positive discourses about autistic identity include those by the neurodiversity activist Sinclair^6^ who argued that separating “autism” from “self” is a false dichotomy and that autism is “different” (not more or less than nonautistic ways of being). One danger in the endeavor of the separation of “autistic” traits from generalized personality traits is that the work may reify autism as an entity separate from self.

### Limitations

Our study has some limitations. We adopted a sampling frame to be as inclusive as possible. However, there was a lack of severely intellectually and language-impaired participants. Eliciting the opinions of these groups would require a different approach, perhaps observational.^51^

### Future research

An area for future research could be to examine whether adults with other neurodevelopmental conditions report that their conditions bring advantages, and if so, how. Whether autism (and other neurodevelopmental conditions) may be reconceptualized from solely deficit-based to a more nuanced view is a promising avenue of enquiry.

## Appendix

### Appendix 1: Topic Guide/Interview Schedule

#### Preamble for Interviews

Hello, **Participant**. I want to spend a few moments introducing myself and telling you why I am here doing this interview with you today. My name is **xxxxx** and I am a researcher from the University of Exeter, I am working on a project investigating autism, and at the moment we want to learn more about your background, your abilities, your experiences of services, and your views on this. I want to learn more about these issues from your perspective.

So, for around the next hour or so I want to ask you a series of questions that have been designed to encourage you to talk about any positive or beneficial aspects of autism, and your experiences although there will be some questions that focus on other aspects of autism too. My hope is that our research can use some of the things you say today to help people—for example by advising service providers what you would recommend for other people. Before I start to record this interview, I want to make sure that you are fully aware of a few things.

You are free at any point to stop this interview—for any reason—and you are not required to tell me why you want to stop.

You are completely free to refuse to answer any question. Again, you do not need to provide any explanation as to why, all you need to do is tell me “I don't want to answer that question” or “Please can we move on?”

So if you feel uncomfortable or uneasy about anything during this interview—whether it be something to do with the questions or the layout of the room—please say so and I will do whatever I can to fix this. Just say “Can we stop there?” (Get them to say it to check they understand.)

We have prepared a consent form for everyone involved in this project to read and sign. The idea is that we read through this together and I can explain what each part of it means. Do not worry, there is not anything difficult or complex you need to worry about—these are all standard things for a research project. So is it okay if I read through this with you now? **Read the information/consent form to the participant, ask them to sign if they are clear about content, and happy to.**

Thanks for that! Right then, as mentioned in the form we just signed, everything here will be confidential. All identifying names here will be removed in any reports. So will it be okay if I start the recorder? I do not want to miss anything that we discuss….

##### Start recorder and lapel MIC

The interview starts off quite boring with yes/no answers, but it gets better later on when it moves to your experiences, and most people enjoy talking about their experiences, so it should be enjoyable.

#### Demographic/Introduction Questions

I want to start this off by learning more about you. So to begin with, how old are you?

Some assessment of support received

- Where do you live at the moment? If the participants live independently, or what level of support they require
- Who do you live with?

What is your highest academic qualification?

- No qualifications
- School level
- University level

Are you a student, do you have a job, or what is your occupation?

Just to clarify (we will confirm this through provider/carer if appropriate)

- Do you have a medical diagnosis of an autism spectrum disorder?

(If yes) do you know how old were you when you received it? What was the diagnosis?

#### Topic Guide

Much of what we hear about autism focuses on the problems autistic people experience, but today we are particularly interested in learning more about the possible advantages associated with autism.

##### Broad question alternatives

What aspects of yourself do you attribute to your autism?

Are there any aspect of your autism may have been advantageous or beneficial to you?

##### Probes

Has your autism meant that you are particularly good at something?

Are there any aspects of autism that you feel make your life **easier**?

Follow up questions as appropriate—participant may need prompting and have more info than just one positive attribute. Ask participant to give examples of benefits in their home life, work, relationships, job (if applicable), and explore when they found attribute helpful, and in what ways.

Encourage talk about own interests and applications of own skills.
